# Adaptation and preliminary psychometric properties of three self-stigma outcome measures for people living with dementia

**DOI:** 10.1186/s12877-020-01983-0

**Published:** 2021-01-09

**Authors:** Jem Bhatt, Charlotte R. Stoner, Katrina Scior, Georgina Charlesworth

**Affiliations:** 1 Research Department of Clinical, Educational and Health Psychology, University College London, London, UK; 2 Research and Development, North East London Foundation Trust, Essex, UK

**Keywords:** Stigma, Alzheimer’s, Cognition, Outcomes, Reliability, Validity

## Abstract

**Background:**

A diagnosis of dementia presents individuals with both social and psychological challenges but research on self-stigma in dementia has been largely confined to qualitative approaches due to a lack of robust outcome measures that assess change. The Stigma Impact Scale (SIS) is the most commonly used measure of self-stigma in dementia but its suitability as a tool to assess change in a UK population is unclear. Thus, the aim of this study was to identify, adapt and evaluate the acceptability and preliminary psychometric properties of self-stigma measures for people with dementia for use as measures of change.

**Method:**

A 4-step sequential design of identifying, selecting, adapting and testing psychometric measures as follows: 1) identification of stigma outcome measures through reviewing anti-stigma intervention literature, 2) selection of candidate measures through quality assessment (Terwee criteria) and expert consultation, 3) adaptation for UK dementia population (Stewart and colleagues Modification Framework) 4) testing of adapted measures in people living with dementia (*N*=40) to establish acceptability and preliminary reproducibility (test retest), criterion (concurrent with SIS) and construct (negative convergence with Rosenberg self-esteem scale) validity.

**Results:**

Seven measures were identified from the review, but most were poor quality (Terwee range: 0–4). Three measures were selected for modification: Stigma Stress Scale; Secrecy subscale of the Stigma Coping Orientation Scale; Disclosure Related Distress Scale. Internal consistency and test-retest reliability were acceptable (.866≤α≤ .938; ICC .721–.774), except for the Stigma Stress Scale (α= .643) for which the component subscales (perceived harm, ability to cope) had stronger psychometric properties. Concurrent validity with the SIS was not established (*r*<.7) although there were significant correlations between total SIS and perceived harm (*r*=.587) and between internalized shame and secrecy (*r*=.488). Relationships with self-esteem were in the hypothesized direction for all scales and subscales indicating convergent validity.

**Conclusion:**

Stigma scales from mental health are not readily adapted for use with people with dementia. However there is preliminary evidence for the acceptability, reliability and validity of measures of perceived harm, secrecy and stigma impact. Further conceptual and psychometric development is required.

## Background

Dementia is a syndrome that comprises a collection of symptoms characterised by a decline in, and ultimately a loss of, cognitive functions such as decision-making, attention and awareness, planning, inhibition, learning, memory and language. Dementia is categorised as a major neurocognitive disorder in the Diagnostic and Statistical Manual of Mental Disorders 5th Edition [1]. There are a number of underlying neuropathologies or neurodegenerative illnesses that ultimately lead to dementia, for example Alzheimer’s disease and vascular disease.

Receiving a diagnosis of dementia presents individuals with both social and psychological challenges where stigma can be a pivotal and powerful negative force shaping people’s experiences [2]. The recent World Alzheimer Report on Attitudes to Dementia concluded that people living with dementia are stigmatised across many different domains, such as social life, finances, housing, healthcare, intimate relationships, making or keeping friends and being treated unfairly by children or family [3].

### Self-stigma and its impact

Self-stigma, also referred to as internalized stigma, is a cognitive process whereby an individual internalises negative stereotypes and prejudice related to their stigmatised identity [4]. There is a well evidenced connection between mental health difficulties and the experience of self-stigma which in turn was associated with lower levels of empowerment, self-esteem, hope, self-efficacy, symptom severity, treatment adherence, social support and quality of life [5, 6]. Self-stigma has also been linked to negative consequences of concealing a diagnosis (e.g. anxiety and depression) and withdrawal from health services [5, 7, 8].

People with dementia have also been found to be at risk of self-stigma. A recent systematic review found self-stigma to be associated with anxiety and depression, personal control, self-esteem, social support and activity participation [9–11]. More specifically, self-stigma has lasting negative consequences for people living with dementia such as withdrawing from everyday activities or interactions, delays in help-seeking, loss of confidence or feeling inferior [12–16].

### Quantifying self-stigma

The measurement of self-stigma in mental health is complex, with a recent review of 57 empirical papers documenting five self-stigma outcome measures for people with a mental health diagnosis [17]. Authors of the five self-stigma measures reported content validity, however no or little detail was given on other important psychometric properties including internal consistency and convergent validity. A further systematic review examining the efficacy of psychosocial self-stigma interventions for people with schizophrenia-spectrum diagnoses identified six self-stigma measures from 12 studies but, again, these measures were subject to limitations including no of sensitivity to change in seven randomised controlled trials (RCTs). Collectively, both systematic reviews concluded that further refinement of self-stigma measures in line with reliability and validity criteria, careful cultural considerations and condition-specific adaptation with those who have lived experience of the condition are necessary avenues for future research [17–19].

### The stigma impact scale

The Stigma Impact Scale (SIS) is the only self-stigma scale that had been previously tested in populations living with dementia [11]. It has three subscales (social rejection, social isolation, internalized shame) and was based on the adapted Multidimensional Model of Perceived Stigma, which was used to explain self-stigma experienced by people living with Alzheimer’s and Parkinson’s disease [20]. Recent testing of the SIS in people living with dementia suggests an association between decreased levels of self-esteem and increased levels of internalized shame and social isolation, speaking to the inverse relationship between self-esteem and stigma concepts which has been found in other mental health conditions [10, 21].

### Rationale for study

The intervention that informed present work, Honest, Open, Proud (HOP) is a group-based psychosocial intervention delivered over three sessions to help people with mental health difficulties consider disclosing stigmatised identities (e.g. a diagnosis of schizophrenia) across settings [21, 22]. HOP has recently undergone adaptation to support people living with dementia [23], however, there are currently no ‘gold standard’ outcome measures available to evaluate the effectiveness of psychosocial stigma reduction interventions in dementia, with previous studies criticised for not reporting psychometric properties of measures used or using non-standardised outcomes [11, 24]. As such, there is a need for standardised and psychometrically robust outcome measures developed specifically to evaluate self-stigma for people with dementia. The aim of the current study was to identify, modify and test the feasibility and psychometric properties of existing stigma instruments used in mental health research for use with people living with dementia.

## Methods

### Design

The design of the current study was a sequential process with four-stages consisting of identifying, selecting, adapting and testing psychometric measures.

### Stage 1: review of HOP outcome measures

A review of Honest Open Proud (HOP) intervention studies was conducted to identify instruments that have been previously used in peer reviewed journal articles up until December 2018. Instruments were only included if: the focus of the measure was self-stigma as defined by Corrigan and Colleagues [25]; the instrument had been used as an outcome measure in the evaluation of a HOP intervention; intervention studies were published in peer reviewed academic journals. Instruments were excluded if the focus of the measure was on constructs not applicable to dementia, for example symptomatic recovery.

### Stage 2: measure selection

Measures were selected using a combination of psychometric quality and research team appraisal.

#### Psychometric quality appraisal

The instruments identified were appraised for psychometric quality using guidance by [19], which has been used in previous research to establish the quality of psychometric instruments [26–28]. A focussed search for journal articles describing the development of each identified measure was conducted and each was appraised across seven domains: a) content validity, b) internal consistency, c) construct validity, d) reproducibility (in two parts: agreement and reliability), e) responsiveness, f) floor and ceiling effects and g) interpretability.

Any instrument could score a maximum of 2 per domain and a minimum of 0. Further details of scoring procedures are described in [26]. Overall quality appraisal scores were calculated by summing the scores for each domain, with a potential score range between 0 and 18. Labels were assigned to interpret the quality of the instruments based on [26] where instruments that scored 0–4 were categorised as ‘poor’ quality, 5–9 as ‘moderate’ quality, 10–14 as ‘good’ quality, and 15–18 as ‘very good’ quality.

#### Research team appraisal

In addition to the quality appraisal criteria, two experts also appraised instruments, one an expert in stigma and disability (KS), the other an older adult’s expert with specialist knowledge in the measurement of psychological constructs (GC). Collectively, decisions were made to include instruments if all three of the following criteria were satisfied:

1. Instrument did not require significant changes to language that might invalidate previous psychometric findings (e.g. stereotypes and language used would be similar for a UK population);
2. The instrument was deemed acceptable and relevant for a person living with dementia;
3. The instrument could serve as a feasible outcome measure for an anti-stigma intervention for people living with dementia (“Who to tell, how and when?”)

### Stage 3: adaptation and modification

#### Consultation with experts

##### Research Experts

Five expert researchers in the field of dementia research (1- dementia prevention assessment and intervention, 2- behaviour change and intervention fidelity, 3 - positive psychological outcomes and psychometrics, 4 - mixed methods research understanding the impact of chronic health conditions, 5- psychological support for people living with dementia and family carers) were asked to review the instruments on an item by item basis. The items were sent to each expert in a word document with instructions to indicate which items were relevant to people living with dementia based on their suitability and acceptability. Once all research experts indicated their views, they were collated in an excel spreadsheet.

##### Lived Experience Experts

A second expert group was made up of lived experience experts (people living with dementia and carers) involved in a patient and public involvement (PPI) capacity. PPI members were split into three sub-groups of approximately 2–3, with each group supported by one researcher. The instructions were to perform a card-sorting task where all items of the selected instruments were presented on strips of paper in no particular order and had to be sorted into two envelopes labelled “acceptable” and “not acceptable”. PPI members were informed that, in order for an item to be deemed acceptable, they must feel that it is understandable, relevant and that a person living with dementia would be able to answer the question. A round-robin technique was used to elicit thoughts and discussions on items from each member of the sub-groups. This methodology [29] allows for all group members to communicate a position rather than the acceptability of items being determined by a dominant personality. The card-sorting task was designed so that each item was reviewed at least twice by two different groups.

#### Measure modification framework

A measure modification framework [30] was used to incorporate modifications from consultation with two expert groups [29, 31]. In the event that expert groups had conflicting feedback about the instruments, discussions between authors were used to resolve this until a conclusion was reached. The Modification Framework [30] increased the likelihood that adaptations to the psychometric measures would lead to items with comparable meanings, reliability and validity to that of the original measures. Three types of modifications were used based on the above expert consultation: (1) drop dimension (a dimension (subscale) is omitted), (2) drop items (items are removed from an existing scale) and, (3) modify items (substituting a term or modifying wording without changing meaning).

### Stage 4: pilot testing

#### Participants

Participants were included if they: (1) were an adult over the age of 18, and (2) had a primary progressive diagnosis of dementia. Participants were excluded if: (1) they had a chronic, terminal medical condition of which they were in the later stages, (2) they had a significant sensory impairment that could not be compensated for and precluded participation, and (3) they lacked capacity to consent to the study according to established guidelines [32, 33]. Ethical approval for this research was granted by the University College London Research Ethics Committee (Project: 11501/002).

Participants were recruited via three avenues: (1) researchers contacted participants who declared an interest or were matched to the study criteria on the Join Dementia Research (JDR) database, (2) self-identification where participants had heard about the research and expressed an interest in taking part (e.g. via social media and advertisements placed in local community buildings and shops), and (3) through outreach activities carried out by the researchers such as attending dementia groups (e.g. Alzheimer’s Society localities).

#### Measures

Selected and modified measures from steps 1 to 3 were administered alongside:

*Stigma Impact Scale (SIS)* to test for concurrent validity*.* All 21 items were rated from 1 (‘strongly disagree’) to 4 (‘strongly agree’) with the addition of 0 for ‘not applicable’ items across four subscales, namely, social rejection (9 items, e.g. “I feel others avoid me because of my impairment”), internalized shame (5 items, e.g. “I feel others think I am to blame for my impairment”) and social isolation (7 items e.g. “I feel set apart from others who are well”). As per previous research, the financial insecurity subscale was excluded [34–36].

*Rosenberg Self-Esteem Scale (RSES)* to test for convergent validity [37], which consisted of 10 items rated from 1 (‘strongly disagree’) to 4 (‘strongly agree’) measuring an individual’s beliefs and attitudes of themselves (e.g. “On the whole, I am satisfied with myself”).

#### Procedure

Potential participants were given a study information sheet and at least 24 h to consider participating before consent was sought. Participation methods were either independently online or face-to-face data collection where one researcher (the lead author or an MSc student) conducted home visits. For the latter, participants completed the measures independently or adjustments were made if this was not possible, for example the researcher would support a participant by ticking their preferred response or providing hard copies with larger font format. Qualtrics (Qualtrics, Provo, UT) was used for online data collection, where a participant accessed the participant information sheet, screening questions, consent form and study measures through a survey link. During face-to-face data collection, these documents were presented to participants.

A subsample of participants were asked to complete the study instruments one to two weeks later (T2) in the same format in which they had completed them initially (T1).

#### Statistical analysis

The Statistical Package for Social Sciences (SPSS, Version 26) was used for data input and analysis.

#### Acceptability and Suitability

Acceptability and suitability were ascertained using completion rates, time taken to complete T1 and floor and ceiling effects. This was due to the premise that a more acceptable and suitable instrument would yield high completion rates, have similar times of completion across measures and no floor or ceiling effects would be present. If 15% of participants achieved the highest or lowest possible scores, floor and ceiling effects were considered significant. Researchers who conducted home visits took field notes on their experience of completing the instruments to understand the acceptability and suitability of the instruments.

#### Reliability

##### Internal consistency

The internal consistency for each scale and subscales was assessed using Cronbach’s Alpha. A value for alpha ≥ 0.7 is considered acceptable [38].

##### Test Retest

Stability was assessed through an Intraclass Correlation Coeffcient (ICC) analysis using a two-way random effect model. ICC figures ≥.70 or above indicate stability [19, 39].

#### Validity

##### Concurrent validity

A Pearson Product-Moment Correlation Coefficient (Person’s *r*) was used to assess concurrent validity against the SIS. A correlation of ≥.70 was considered an indication of good concurrent validity [19].

##### Convergent validity

The RSES was used to assess convergent validity as self-esteem has been previously negatively correlated with stigma experience (e.g. application of self-stigma and secrecy). It was hypothesised that a low to moderate negative correlation between self-stigma and self-esteem would be documented as per previous research [20]. If at least 75% of the results are in accordance with this hypothesis, this demonstrates adequate convergent validity [19].

## Results

### Stage 1: review of HOP outcome measures

Seven stigma instruments were identified from three HOP intervention studies: Perceived Devaluation Discrimination Questionnaire (PDDQ; [40]); Coming Out With Mental Illness Scale (COMIS; [41]); Stigma Stress Scale (SSS; [42]); Self-Stigma Of Mental Illness Scale (SSMIS; [43]); Stigma Coping Orientation Scale (SCOS; [44]); Internalized Stigma Of Mental Illness (ISMI; [45]); Disclosure Related Distress Scale (DRDS; [46, 47]).

### Stage 2: measure selection

#### Quality appraisal

None of the identified measures reported information on reproducibility-agreement and responsiveness (see Table 1). Internal consistency findings using Cronbach’s alpha (between > 0.70 or < 0.95) in the absence of a factor analysis was reported for all measures apart from the SCOS. Criterion validity and floor and ceiling effects were only reported for the ISMIS [45]. Content validity was adequately reported only for the COMIS, SSMIS, ISMI and SIS with a clear description of the measurement aim, target population, concepts being measured, item selection. The SCOS did not report any target population involvement in item selection. Construct validity was adequately reported for the ISMI but not for the SSMIS and all other measures only partially met the criterion (as less than 75% of hypotheses were confirmed despite adequate design and methods). Information on reproducibility reliability was adequately reported for only the SSMIS and ISMI. Interpretability was adequately reported for the PDDQ and SCOS however, only partially reported for COMIS, SSS, ISMI and SIS (no definition of minimal important change or absence of at least four subgroups). No interpretability findings were reported for SCOS. It was not possible to appraise the psychometric quality of the DRDS [42, 43] as the scale is an unvalidated measure previously used as a screening tool for HOP with no reported psychometric properties [46].

**Table 1:** Quality and expert appraisal of stigma instruments

		Psychometric Quality Appraisal^d^										Expert Appraisal			
Scale	Reference	Content Validity	Internal Consistency	Criterion Validity	Construct Validity	Reproducibility		Responsiveness	Floor/Ceiling Effects	Interpretability	Total	Criterion 1^a^	Criterion 2^b^	Criterion 3^c^	Decision
						Agreement	Reliability								
Perceived devaluation and Discrimination Questionnaire (PDDQ)	Link, (1987) [40]	0	1	0	1	0	0	0	0	2	4	No	Yes	Yes	Excluded
Coming Out Proud with Mental Illness Scale (COMIS)	Corrigan et al. (2010) [41]	2	1	0	1	0	0	0	0	1	5	No	No	No	Excluded
Stigma Stress Scale (SSS)	Kaiser et al. (2004) [48]	0	1	0	1	0	0	0	0	1	3	Yes	Yes	Yes	Included
	Rüsch et al. (2009) [42]	0	1	0	1	0	0	0	0	1	7				
Self-stigma of mental illness scale (SSMIS)	Corrigan et al. (2006) [43]	2	1	0	0	0	2	0	0	0	5	Yes	Yes	Yes	Included
Stigma Coping Orientation Scale (SCOS)	Link et al. (1989) [49]	0	0	0	1	0	0	0	0	1	2	No	Yes	No	Excluded
	Link et al. (2002) [44]	2	0	0	0	0	0	0	0	2	4				
Internalised stigma of mental illness (ISMI)	Ritsher et al. (2003) [45]	2	1	1	2	0	2	0	1	1	9	No	Yes	No	Excluded
Stigma Impact Scale (SIS)	Fife & Wright, (2000) [50]	2	1	0	1	0	0	0	0	1	5	Yes	Yes	Yes	Included
	Burgener & Berger, (2008) [20]	1	1	0	1	0	0	0	0	1	4				

^a^Measure would not require significant changes to language that would require invalidating previous psychometric findings

^b^Measure is acceptable and relevant for people living with dementia

^c^Measures serves as a feasible outcome measure for a disclosure decision-making intervention for people living with dementia (“Who to tell, how and when?”)

^d^modified from Terwee et al. (2007) [19], for scoring see Stansfeld et al. (2017) [26]

#### Expert appraisal

The SSS, SSMIS and SIS met all expert appraisal criteria (see Table 1). The PDDQ, ISMI, COMIS, SCOS would have required significant changes that would invalidate previous psychometric findings, such as mentions of symptomatic recovery throughout (‘going back to work after recovery’) and item stems across subscales that were not deemed relevant or acceptable for a UK population of people living with dementia (“I came out of the closet”; “I stayed in the closet”; “I will come out of the closet”; “I stay in the closet”) and the lack of transference of stereotypes from mental health to dementia. The COMIS was appraised as being the only measure that would not be accessible and relevant for people living with dementia. The COMIS, SCOS and ISMI were deemed unsuitable to serve as feasible outcome measures for a disclosure decision-making intervention for people living with dementia. This was because the COMIS dichotomised disclosure between ‘coming out’ and ‘staying in’ the closet rather than acknowledging the stages in-between (e.g. selective disclosure). The SCOS had only one subscale containing relevant concepts to disclosure, whilst the others were psychiatric treatment based.

### Stage 3: results of adaptation and modification

Lack of appropriate or relevant language for people living with dementia, cognitive burden of completion and the inclusion of items around recovery were the main issues with the identified measures. It was necessary to drop all dimensions on the SSMIS and four subscales on the SCOS to leave only the secrecy subscale of the SCOS (SsSCOS). All dimensions on the SIS and SSS were retained. Item removal was necessary for the SsSCOS where two items were not relevant for people living with dementia “In order to get a job a former mental patient will have to hide his or her history of hospitalisation” and “you believe that a person who has recovered from mental illness earlier in life should not tell other people about it”. Item removal was necessary for the DRDS where the second item of the scale referring to employer/teacher disclosure was deemed irrelevant and removed. Consequently, the first item was divided in two, where the first item asked about disclosure to friends and the second to family. The DRDS items read as follows “In general how comfortable would you feel talking to [item one: a friend; item two: a family member] about dementia, for example, telling them you have a dementia diagnosis and how it affects you?”. Item modifications were made on the SSS, SsSCOS and DRDS to remove “mental illness” references to “dementia”. In the SSS, the term “prejudice” was replaced with “stigma” on the premise that prejudice and stigma are interchangeable terms yet stigma is the most colloquially appropriate. For the SIS, the term “dementia” was inserted into the instructions to be used interchangeably with “impairment”. See Table 2 for modification and adaptation summary.

**Table 2:** Modifications and adaptation of selected stigma instrument

	Stigma Stress Scale	Self-stigma of Mental Illness Scale	Secrecy Scale	Stigma Impact Scale
**Scale Description**	8 item Likert scale from 1 •(strongly disagree) to 7 (strongly agree). Four items are design to measure the harm caused by stigma and four focus on the impact of stigma on one’s resources to cope with such harm.	4 subscales answered on a 9-point likert scale representing: **awareness** of stereotypes, **agreement** with stereotypes, **apply**ing stereotypes to self and suffer **harm** from self-applied stereotypes. Each subscale has five items	9 items are answered on a 4-point Likert scale from 1 (strongly disagree) to 4 (strongly agree). Assess the extent to which an individual endorses concealment as a means of avoiding stigma related rejection.	21 items are rated from 1 (‘strongly disagree’) to 4 (‘strongly agree’) with the addition of 0 for those items participants found ‘not applicable’. The scale has four subscales, namely, social rejection (9 items), internalised shame (5 items) and social isolation (7 items).
**Comments from lived experience experts**	Just call it stigma rather than prejudice—in an UK population stigma is more colloquial than prejudice	The term “less confidence” should be used rather than “less respect”	No comments	No comments
**Comments from research experts**	Define prejudice in the instructions Item 6 wording is complicated Items 7 & 8 similar Supplement challenges for the word demands	Using “most people” and “the public” to describe the same thing is confusing. The perspective change between subscales was problematic in the past. This scale relies on stereotypes of mental health, therefore these also need to be relevant to dementia	Removal of recovery and employability item. Issues with the term impairment – maybe use “diagnosis”	Change the use of the word “impairment”, for example replace with “dementia”
**Drop dimension**	None	All dimension dropped	Dropped four of five dimensions from the original Stigma Coping Orientation Scale to leave only the secrecy subscale	None
**Items Removed**	None	None	Items 6 and 8 removed	None
**Item** **Modification**	“people with mental illness” changed to “people living with dementia” . “prejudice” changed to “stigma” .	None	“mental illness” changed to “dementia” for the purposes client group adaptation	None
**Other** **Modification**	“Prejudice” was replaced with “stigma” in the instructions, the definition was left unchanged on the premise that prejudice and stigma are interchangeable terms yet stigma is the most colloquially appropriate	None	None	The term “dementia” was inserted into the instructions to be used interchangeably with “impairment”. The instructions read “dementia or neurological impairment …”

### Stage 4: pilot testing

#### Sample characteristics

Forty-One people living with dementia met the eligibility criteria and provided informed consent to take part in this study. One participant who took part online was excluded due to large amounts of incomplete data. Eighteen participants took part online and 22 participants completed the study during face-to-face visits. Sample characteristics are summarised in Table 3. Three participants were unable to remember the nature of their diagnosis. The majority of participants were native English speakers of ‘white’ ethnic background with one participant declining to complete this question.

**Table 3:** Participant characteristics and demographics

Sociodemographic Variables	M *(SD) or N*
Age, years	72.40 *(10.61)*
range	56–95
Months since diagnosis	45.20 *(33.10)*
range	2–120
Sex (M/F)	20/20
Type of dementia	
Alzheimer’s Disease	21
Vascular Dementia	7
FTD (behavioural variant)	1
Lewy Body	1
Mixed	6
Not disclosed/Unknown	4
Ethnicity	
White	36
Other Ethnic Group	3
Not disclosed	1
No. Living alone	
Yes	13
No	27
Employment status	
Employed	5
Retired	30
Other	4
Not disclosed	1
English as first language	
Yes	37
No	3

#### Acceptability and suitability

The reported scores on the SSS, SIS and SsSCOS were normally distributed, with low levels of missing data. A Little’s Missing Completely At Random (MCAR) was non-significant for each measure (*p* = 1.00) indicating data were missing completely at random (MCAR) and therefore mean imputation at an item level was appropriate to deal with missing data [51, 52].

Time taken was recorded for a small sample of face-to-face participants who took a mean of 43 min (*n* = 7) to complete the measures at T1. The time taken for completion ranged from 15 to 60 min. No floor or ceiling effects were identified as the percentage of participants scoring the lowest or highest possible scores on an instrument was lower than 15%.

Field notes were collected during 14 of the 22 home visits carried out for face-to-face data collection. Three participants found the response categories of the SsSCOS challenging for items that were a double negative (item 1 and 4) but also because often the response was dependent on who the participant had in mind (e.g. item 7 of SsSCOS). For items that required more thought, participants read aloud items as questions for themselves with each response category (e.g. Do I agree that [item wording]) or included the item in a sentence with response categories (e.g. I agree that [item wording]), to establish a level of agreement and disagreement and whether it was strong or not. The scales were presented in tables with items on each row and response categories on each column. One participant found it difficult to align the column and rows to tick the appropriate response box.

Two participants found the phrase ‘stigma against people living with dementia’ (SSS) confusing due to being unsure whether the item was referring to themselves as a person living with dementia, to others with dementia but not themselves, or to people living with dementia more generally. One participant found item 21 of the SIS (“changes in my appearance have affected my social life”) difficult to relate to dementia.

#### Internal consistency

The SIS (α =.906) and SsSCOS (α =.864) had acceptable internal consistency but the SSS (α =.643) did not. The Cronbach’s alpha values for all subscales were acceptable with the exception of the SIS subscale of internalized shame (α = .614) which fell below the cut-off for acceptability and the internal consistency was not improved through item removal.

#### Test re-test reliability

ICC_agreement_ estimates and their 95% confidence intervals were calculated using data from 25 participants who completed both T1 and T2. Reliability of the majority of measures between T1 and T2 was moderate (see Table 4 for exceptions).

**Table 4:** Summary of descriptive, reliability and validity statistics

		Floor and Ceiling				Reliability				Validity (Pearson’s R)				
	M *(SD)*	Min	Max	Lowest Score (%)	Highest Score (%)	Internal consistency (α)	Test - Retest			Concurrent				Convergent
							ICC	CI^a^						
										SIS Total	SR	IS	SI	
								Lower	Upper					
SSS	−7.73 *(10.37)*	−24	16	5	0	.643	.721	.467	.866	.525**	.441*	.160	.590**	−.475*
SSS Harm	15.73 *(8.16)*	4	28	15	12.5	.938	.864	.717	.937	.587**	.499*	.177	.654**	−.295*
SSS Cope	23.47 *(4.02)*	12	28	5	22.5	.866	−.199	−.507	.294	−.161	−.124	−.055	−.193	.186
SsSCOS	1.83 *(0.64)*	1	3	10	0	.864	.746	.503	.880	−.001	−.175	.488*	−.015	−.0.32,
SIS	42.54 *(12.88)*	21	79	0	0	.906	.774	.550	.894					−.587**
SR	17.24 *(6.90)*	7	36	0	2.5	.868	.707	.435	.860					−.416*
IS	8.60 *(2.84)*	5	16	0	0	.614	.518	.158	.759					−.483*
SI	16.07 *(5.53)*	7	28	0	2.5	.869	.841	.674	.927					−.600**

*p <.05 **p<.001

#### Concurrent validity

The SSS total was positively correlated with the SIS, however, the correlation coefficient was below the necessary cut-off to demonstrate satisfactory concurrent validity SIS (*r* = .525, *p* <.001). The perceived harm subscale of the SSS and the SIS total were positively correlated but the ability to cope subscale of the SSS did not correlate with the SIS total. This may be because they quantify conceptually different components (ability to cope vs social and psychological impact of stigma). The perceived harm subscale of the SSS was positively correlated with the social rejection and social isolation subscales of the SIS but not the internalized shame subscale. The ability to cope subscale of the SSS did not significantly correlate with the SIS subscales of social rejection, internalized shame or social isolation. The SsSCOS and the SIS total (*r* = −.001, *p* >.05), social rejection and social isolation subscales were not significantly correlated. The SsSCOS was positively correlated with the internalized shame subscale of the SIS.

#### Convergent validity

In line with predictions, the overall SSS (*r* = −.475, *p* <.05) and SIS (*r* = −.587, *p* <.001), including all subscales with the exception of ability to cope subscale, were negatively correlated with the RSES. Correlations were within the predicted range of low-moderate with the exception of the perceived harm subscale. The SsSCOS (*r* = −.0.32, *p* >.05) did not significantly correlate with the RSES, lack of significance was not in line with predictions or previous research yet the direction of the correlation was.

## Discussion

This is, to our knowledge, the first study to report the acceptability, suitability and psychometric properties of self-stigma measures for people living with dementia. The results of the small-scale pilot suggest that the Stigma Stress Scale, secrecy subscale of the Stigma Coping Orientation Scale, and Stigma Impact Scale are acceptable for use in a UK population of people living with dementia. Almost all measures had moderate test-retest reliability, suggesting they may be suitable for use as outcomes measures (baseline versus follow-up), and all measures except the SSS total, and the internalized shame subscale, had good internal consistency. Concurrent (criterion) validity with the Stigma Impact Scale was tested for, but did not reach criterion for any correlation. Convergent (construct) validity was established with self-esteem with all correlations in the expected direction.

### Findings in the context of existing research

The link between the appraisal of stigma as harmful (perceived harm subscale of the SSS) and social rejection and isolation was supported in the current study. However, the absence of a correlation between perceived harm and internalized shame was not predicted as previous mental health literature has found that internalized shame plays an integral role in shaping stigma experiences [42, 53]. The SSS may not adequately measure the stigma stress appraisal process for people living with dementia. Further, the internal consistency of the SSS was improved when the subscales were kept separate rather than as one overall score.

The secrecy subscale of the SCOS only correlated with the internalized shame subscale of the SIS, indicating secrecy may be associated more with cognitive components of self-stigmatisation than the more social and overt aspects (social rejection and isolation). Measuring levels of secrecy, therefore, may be a way of operationalising internalized shame rather than measuring the appraisal of stigma (SSS).

The current study found significant negative correlations between the SIS, all three subscales and the RSES, whereas previous work was only able to find this for the internalized shame subscale [20]. The relationship between self-stigma and self-esteem is well documented in mental health stigma research, but less so in dementia. The current study, therefore, evidences the similarly important role of self-esteem in self-stigma for people living with dementia.

The internalized stigma of mental illness scale (ISMI) was excluded at the stakeholder consultation stage of the current study however it has been a popular measure for use in stigma reduction interventions for mental health (for a review see Wood et al., 2016). Although the ISMI had the highest quality rating of all identified measures, the content would have required significant changes for use in a population of people living with dementia. This speaks to the importance of acknowledging the nuances in the experience of self-stigma between clinical populations. With this in mind, the current study has begun to clarify the potential use of stigma measures in dementia, but efforts to establish specific frameworks (e.g. stress appraisal process in dementia) and theories should underpin the modification process as some measures may perform well in certain clinical populations and not others.

### Methodological problems and limitations

The format of participation (online versus face-to-face) may have affected the results as participants may have been more likely to answer in a socially desirable manner if participation took place in person rather than online. In addition to this, four different researchers were involved in administering the instruments during face-to-face participation, potentially affecting inter-rater reliability. However, all researchers were trained in administration of the outcome measures and all had prior experience of working with people with dementia.

Although overall acceptability was satisfied, some participants felt that response categories were too absolute, where the answer would depend on whom the participant was thinking about at the time. For example, “how comfortable do you feel when talking to a friend about dementia?” depended on the “friend” in question, with some participants noting they had told some but not all of their “friends”. Thus, who the participant chose as the referent may have influenced the responses given. However, this feedback can be used to improve measures in the future.

The SIS was the only instrument available that had an existing evidence base for people living with dementia and was therefore used as the ‘gold standard’ measure to assess concurrent validity. However, the SIS may not truly offer a ‘gold standard’ measure as defined by Terwee and Collegues [19]. For this reason, concurrent validity of the SSS and SsSCOS should be interpreted with caution.

The current study has begun to address the criticisms of previous work, namely the lack of reporting on psychometric properties. However, although previous research has suggested 25 to 40 participants are adequate for preliminary development and piloting measures, [54] further large-scale, quantitative studies are needed to confirm the psychometric properties of the SSS, SsSCOS and SIS.

### Future research

As this study only provided tentative psychometric properties for three self-stigma measures, the next stage is to confirm these properties in a large-scale study and conduct further psychometric analysis to understand each measures sensitivity to change. Further, the various components of self-stigma and how they relate to each other and other mental and physical health concepts should be explored.

Currently, there are no models or frameworks to underpin the investigation of self-stigma in dementia, with little quantitative and qualitative work conceptualizing self-stigma. Work such as this should be established as it will form the basis of quantifying stigma experiences for people living with dementia. In addition, self-stigma in relation to other psychological comorbidities such as depression should be considered in future research.

Some participants were recruited through community groups (e.g. peer support) and others through the JDR database. Although the current study did not aim to quantify the experiences of people living with dementia, rather test the acceptability of doing so, it is important to note that those participants embedded in social groups that have shared experiences of dementia presented very different narratives regarding stigma and dementia to those not embedded in such groups. This may have a significant impact on wellbeing for people with dementia and the relationship between social connectedness, isolation and self-stigma which warrants further attention in future research.

### Conclusion

Three self-stigma measures were identified and adapted using a robust four-stage process in which other identified measures were excluded due to poor quality of psychometrics or lack of relevance to dementia. Measures of secrecy, stigma impact and stress appraisal were acceptable for use in a UK population of people living with dementia. However, the psychometric properties were established using only a small sample. Further psychometric analysis is required before such measures can be implemented in psychosocial research.

## Data Availability

The datasets used and/or analysed during the current study are available from the corresponding author on reasonable request.

## Abbreviations

UK: United Kingdom
SIS: Stigma Impact Scale
RCT: Randomized Controlled Trials
HOP: Honest Open Proud
PPI: Patient and public involvement
JDR: Join Dementia Research
RSES: Rosenberg Self-esteem Scale
PDDQ: Perceived Devaluation Discrimination Questionnaire
COMIS: Coming Out With Mental Illness Scale
SSS: Stigma Stress Scale
SSMIS: Self-Stigma Of Mental Illness Scale
SCOS: Stigma Coping Orientation Scale
ISMI: Internalized Stigma Of Mental Illness
DRDS: Disclosure Related Distress Scale
SsSCOS: Secrecy subscale Stigma Coping Orientation Scale
